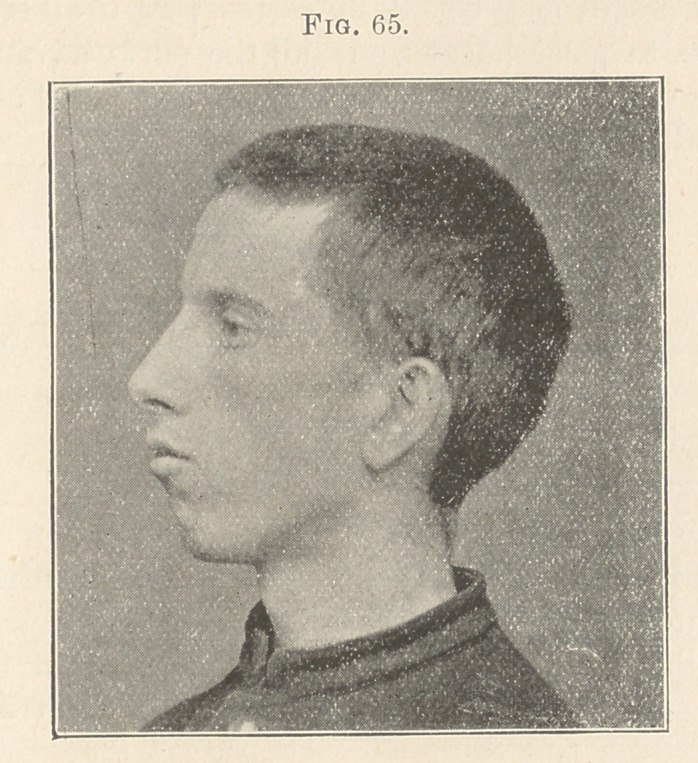# The Degenerate Jaws and Teeth

**Published:** 1897-04

**Authors:** Eugene S. Talbot


### THE DEGENERATE JAWS AND TEETH.[1]

BY EUGENE S. TALBOT, M.D., D.D.S.[2]

(Concluded from page 150.)

MODIFICATION of the V-shaped arch results from modification of the above-named conditions. A difference in the time of eruption of the cuspids, everything else being equal, effects a difference in the space left for their accommodation, and thus partial V-shaped arches (Fig. 55) are found. The key-stone, the cuspid, is not entirely outside or inside of the arch in the partial V-shaped form, but may appear partially crowded out of place. Hence the arch is neither a normal curve nor wholly angular, but unites the characteristics of both. Its lateral diameter is less than that of the normal arch, giving a contracted appearance. Thus a number of varieties of the fundamental forms of the V-shaped arch are formed, differing in degrees of anterior contraction. All of these result from the comparative thinness of the anterior portion of the process offering but little resistance, an abnormal pressure from behind, and the greater strength of the cuspids which cause them to seek room irrespective of the space left for them. When one side of the process near the symphysis is the stronger, thus affording greater resistance, or the pressure from the cuspid is less, that side may maintain its normal relations, while the other may give way to conditions resulting in a V-shaped contraction. The curve will then be broken not at the apex of the triangle, but near it, the incisors will overlap, and when pressure from the cuspid acts on the weaker column it must give way. This results in the semi-V-shaped form (Fig. 56). When the permanent bicuspids erupt under a favorable condition, so that their greatest diameter is in a line with the greatest diameter of both cuspids and first molar, they will be held firmly in place, since the greatest pressure is on this very line. On the other hand, when the bicuspids are erupted after their proper time, while the cuspids progress duly, the cuspids, meeting with no resistance, fall into their proper places, but the bicuspids adapt themselves as best they can to the space left for them, and if the arch of the maxilla does

---

[1] Read in the Section on Neurology and Medical Jurisprudence at the Forty-seventh Annual Meeting of the American Medical Association, held at Atlanta, Ga., May 5 to 8, 1896. Reprinted from the Journal of the American Medical Association, by special request.—[ED.]

[2] Fellow of Chicago Academy of Medicine.

not coincide with that of the crowns they must fall within or without the arch. Now, if the first molar has moved forward, diminishing the space, the bicuspids must erupt either within or without the arch.

To understand why they are generally found within the arch the shapes of the molar and cuspids must be kept in mind. A

Fig. 55.

transverse section of their crowns shows their proximal walls not to be parallel but wedge-shaped, their diameter being greater on the buccal than on the palatal side. When the crowded bicuspid falls within the greatest diameter of these teeth, finding more room within the arch, they naturally slip in the direction of least resistance,—*i.e.*, towards the palate. A local cause for the same condition is found in the fact that the crowns of the bicuspids before their eruption are held between the roots of the temporary molars, and as these form an arch of a smaller circle than that of the permanent teeth, the bicuspids will be found generally inside the arch. From both causes we have an inward curvature which we term the

Fig. 56.

saddle-shaped arch (Fig. 57). It should be noted here that while the V-shaped irregularity is found anterior to the cuspid, the upper incisors are always projecting beyond the lower, the saddle-shaped irregularity is invariably posterior to the cuspid and the bicuspids form an inward curve. The incisors never project. Both forms contract the arch, the V-shaped anteriorly, the saddle-shaped posteriorly. In both forms the forward movement of the first molar is the local cause. When the unfavorable conditions that result in the saddle-shaped arch are not so pronounced we have the partial saddle-shaped arch (Fig. 58). Thus, because of the greater uniformity of the maxilla and of the arch of the crowns there may be more space and the bicuspids may be forced but little out of place, or the molar may move forward but slightly, interfering less with the bicuspids. Sometimes it happens that in trying to adjust themselves to the limited space one bicuspid may be crowded outward and another inward. Sometimes the first bicuspid is in, more fre-

Fig. 57.

quently the second. As has been stated before, this ought not to take place if the greatest diameter of cuspids, bicuspids, and molars were in the same line, but the diameter of one of the bicuspids may be in a line with that of the adjoining tooth while that of the other is not, and then one is pressed along the line of least resistance. As in the case of the V-shaped arch, one side of the mouth may be normal because of the absence of any local condition interfering with the space. One temporary molar may have been extracted, allowing the permanent one to move forward while the other remains in place. The result which follows is an asymmetry of lateral halves termed semi-saddle-shaped irregularity (Fig. 59). How the V-shaped and saddle-shaped arch on one side only may be produced has already been described. How they may be combined on one side remains to be explained. Given thinness of process in the interior part of the mouth, premature or tardy extraction of the cuspid, and there will be a forward movement of the incisors. The development of the cuspid will press the alveolar process inward,

Fig. 58.

thereby contracting the arch, and the tardily erupted bicuspids will adjust themselves to the limited curve, as before stated. In this way the features of the two forms are combined,—that is, a contracted or angular anterior arch and a posterior arch that is more or less concave. The opposite side may be V-shaped, saddle-shaped, or normal (Figs. 60 and 61). Deformities of the dental arch are due first to arrest of development of the jaw, and, secondly, in the nature of the deformity to the order of eruption of teeth which rarely erupt twice alike and is always local or mechanical. From an evolution stand-point these deformities are atavistic. The V-shaped reverts to the reptilian type, the saddle-shaped to the carnivora. Dr. W. C. Barrett, of Buffalo, has frequently called attention to the shape of the anthropoid arch. In the gorilla, the nearest to man in dentition, there is a very distinct approach to the saddle-shape.

Fig. 59.

In the chimpanzee it remains. The orang-outang exhibits a less tendency. The arch of some of the cebidæ very nearly approaches man. It all depends upon the extent of prognathism. When that is reduced the arch appears, and rectangular arrangement of the teeth is lost. Most carnivora exhibit a distinct approach to the saddle-shape. Some felines have a shortening of the jaw, partially obliterating the tendency, but in most canidæ it is quite marked. From the stand-point of comparative anatomy, Dr. Barrett is of opinion that the tendency towards the saddle-shaped arch is a reversion to earlier form.

These are facts which cannot be overlooked, since from the very nature of development and eruption of the teeth they cannot take any other form. The arrangement of the crowns of the cuspid (canine) in the jaw before eruption is such that no matter what the local condition of the jaws or teeth may be, the V- or saddle-shaped dental arch must be produced.

Fig. 60.

In no symptom is degeneracy as evident as in the stigmata resultant on hypertrophy of the alveolar process. This occurs at all ages, but more particularly at the period of development of the permanent set of teeth. The entire alveolar process may become involved (Fig. 62), or only a portion (Fig. 63).

Hypertrophy of the alveolar process is the result of irritation incident upon eruption and the shedding of the temporary teeth and eruption of the permanent teeth.

Laryngologists, rhinologists, and neurologists claim that certain vaults are deformities, when in reality the alveolar process had hypertrophied.

Fig. 61.

Fig. 62.

The jaws, as a whole, owing to an unstable and unbalanced nervous system, are liable to become excessively developed as well as arrested in development. Excessive development of the superior maxilla is evinced by a fulness of the upper lip. In these cases the

Fig. 63.

upper maxilla is too large for the lower and stands out beyond it. The lower may be quite normal. When there is simply a want of proportion between the two jaws it is due to the diminutive or excessive size of one while the other is normal. The criterion in these cases must be the facial angle. The upper jaw is usually in harmony with the skeleton, while the lower jaw depends for its size largely upon function, its size being the result of accident rather than design.

When the upper jaw is normal or smaller than the lower, the extent of its posterior portion is determined by the occlusion of the first permanent molar, which keeps the alveolar process in permanent relation to each other at this point and allows freedom of development in front. If the occlusion is not normal the upper jaw and alveolar process will develop laterally as well as anteriorly.

The teeth of the anterior columns may either stand vertically or they may be turned in towards the lower incisors. The latter defection is produced by the action of the lips. When the cuspids are in their normal position, the upper incisors form a larger arch than the lower, and this permits of their being turned inward, but when the cuspids have moved so far forward that they are not normally interlocked with the lower teeth, the incisors are too crowded to permit this. While the jaws are growing smaller the teeth tend to cause reversion to the original form.

Arrest of development of the superior maxilla is always associated with marked depression at the alæ of the nose, producing the appearance of having been hollowed out from a point at the floor of the orbit to the grinding surface of the lower teeth (Fig. 64). This is the most common type of degeneracy among criminals,

Fig. 64.

and has frequently caused the error of assuming excessive development of the lower jaw, which is normal. And the seeming excess is due to arrest of development of the upper jaw. The lower jaw has in the scale of light gradually grown smaller. Even in some apes it is still large. The changes which have resulted in the negro jaw strikingly illustrate this alteration in size. The lower jaw of the early negro in the South was unusually large. The intermixture of white blood has decreased the size of the jaw until, where there is but a slight admixture of negro blood, the jaw is as delicately shaped as that of the whites. Prognathism of both jaws of the negro arises from the fact that as the lower jaw is much larger in proportion than the upper, the force exerted by the lower against the upper carries the alveolar process and teeth in their formative process forward. This gives prominence to both upper and lower jaws, and its existence is easily demonstrated by examination of our large collection of negro skulls. In the evolution of the race the lower jaw becomes smaller and harmony results in form and size.

Arrest of the lower jaw (Fig. 65) is common among degenerates. This consists of a shortening of the body. Sometimes it is arrested

FIG. 65.

to such an extent that there is apparently no chin. About fifty per cent. of criminals at Elmira, New York, had this deformity.

The following table shows the number of deformities of the jaws and teeth among some of the degenerate classes:

| | JAWS. | | | | | | | | | | TEETH. | | | |
|---|---|---|---|---|---|---|---|---|---|---|---|---|---|---|
| | | | | | | | | | | | | Tubercles of Teeth. | | |
| | Number examined. | V-shaped. | Partial V. | Semi-V. | Saddle. | Partial Saddle. | Semi-Saddle. | Normal. | Arrested development. | Excessive development. | Irregular. | Present. | Absent. | Regular. |
| Criminals at Pontiac, Ill. . . | 465 | 75 | 71 | 3 | 66 | 63 | 16 | 171 | . . | . . | 123 | 13 | 452 | 342 |
| Criminals at Elmira, N. Y. . | 1041 | 381 | 49 | 1 | 157 | 26 | . . | 422 | . . | . . | 220 | 26 | 1015 | 821 |
| Criminals at Joliet, Ill. . . . | 468 | 13 | 79 | 19 | 59 | 92 | 24 | 163 | . . | . . | . . | . . | . . | . . |
| Prostitutes at Chicago, Bridewell . . . . . . . . | 30 | 10 | 17 | 7 | 27 | 10 | 10 | . . | 1 | . . | . . | . . | . . | . . |
| Insane at Dunning, Ill. . . . | 700 | 26 | 47 | . . | 12 | . . | . . | 486 | . . | . . | . . | . . | . . | . . |
| Insane at Kankakee, Ill. . . | 613 | 69 | 107 | 29 | 89 | 105 | 61 | 153 | . . | . . | . . | . . | . . | . . |
| Idiots, imbeciles . . . . . . | 1977 | 129 | 236 | . . | 207 | . . | . . | 1095 | . . | . . | . . | . . | . . | . . |
| Deaf and Dumb . . . . . . | 1935 | 169 | 192 | . . | 203 | . . | . . | 901 | . . | . . | . . | . . | . . | . . |
| Blind . . . . . . . . . . . | 207 | 7 | 9 | . . | 11 | . . | . . | 105 | . . | . . | . . | . . | . . | . . |
| Inebriates* . . . . . . . . . | 514 | 1.5 | 24.4 | 0.3 | 9.3 | 13.2 | 7.7 | 25.4 | . . | . . | . . | . . | . . | . . |

* Per cent.

By carefully studying the atavistic conditions herein noted, so common among the degenerate classes, the concrescence and differentiation theories, as advanced by Magitot and Cope and Osborn, in the evolution of the teeth of man, are proved to be identical.

In conclusion, it may be mildly stated that if all structures are so affected as to cause harmony in all the parts, advance in evolution is going on, but if such conditions exist as noted above, degeneracy is resulting.[1]

[1] The illustrations for this article are taken from the following works: The Dental Cosmos, Diseases and Injuries of the Teeth (Morton and Smale), The Osseous Deformities of the Head, Face, Jaws, and Teeth (Talbot), with a number of original cuts.

## Figures and Tables

**Fig. 55. f1:**
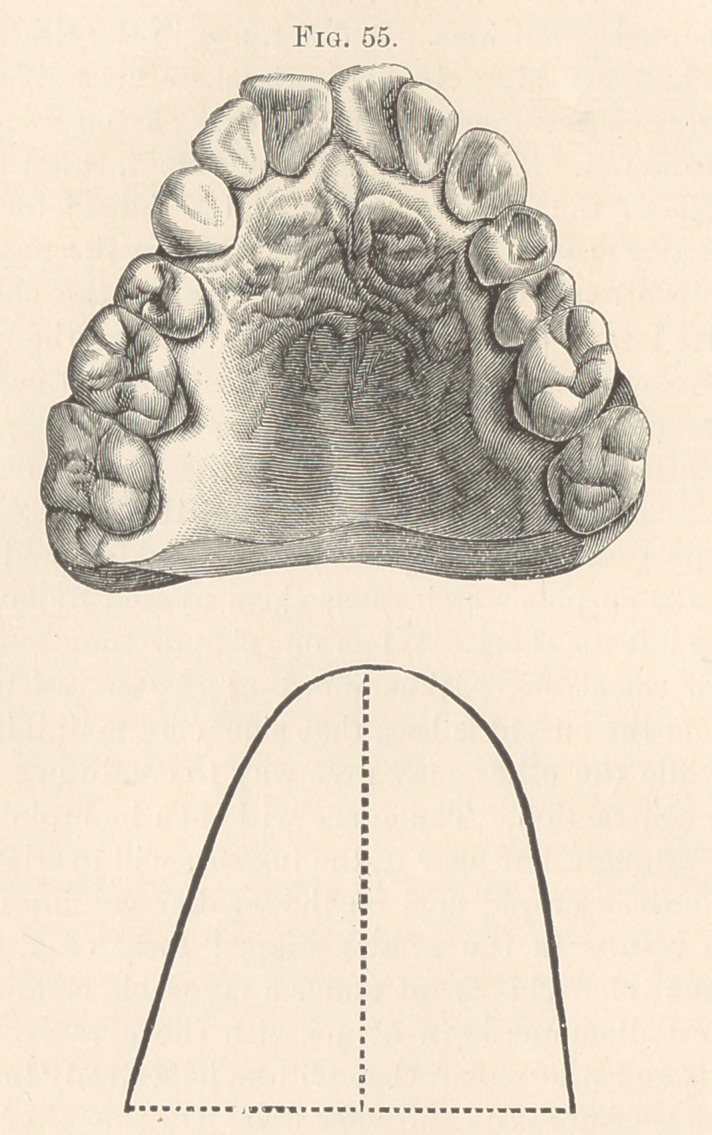


**Fig. 56. f2:**
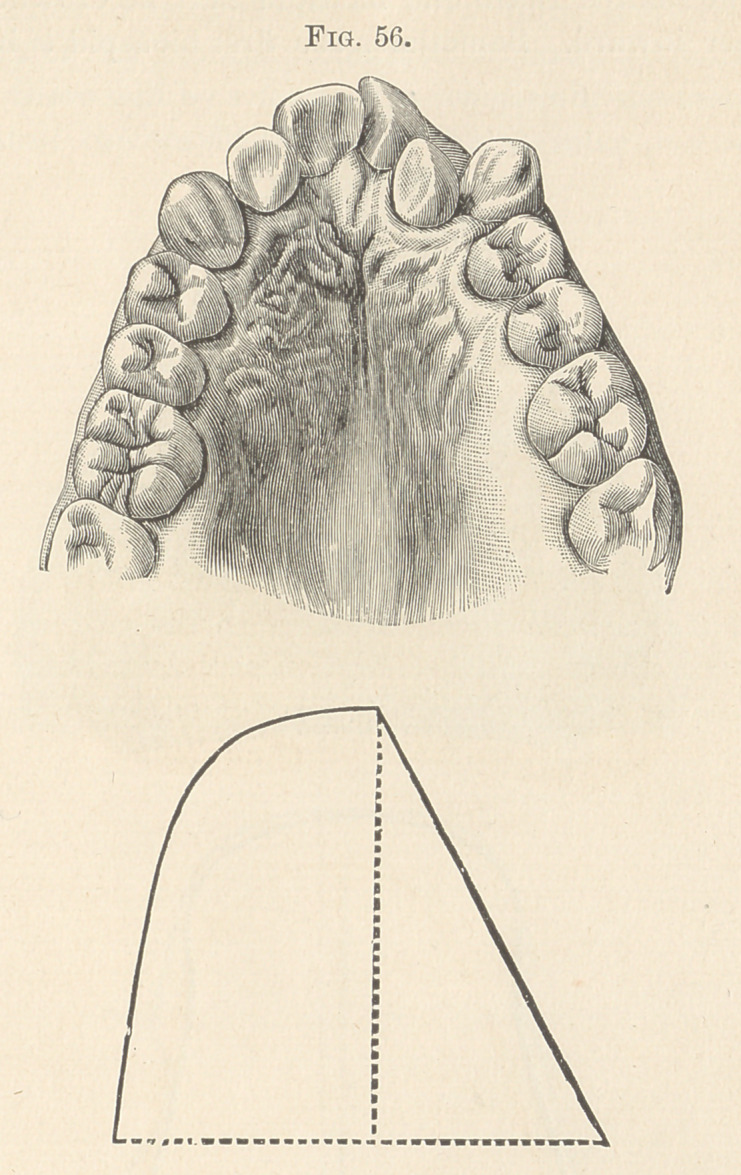


**Fig. 57. f3:**
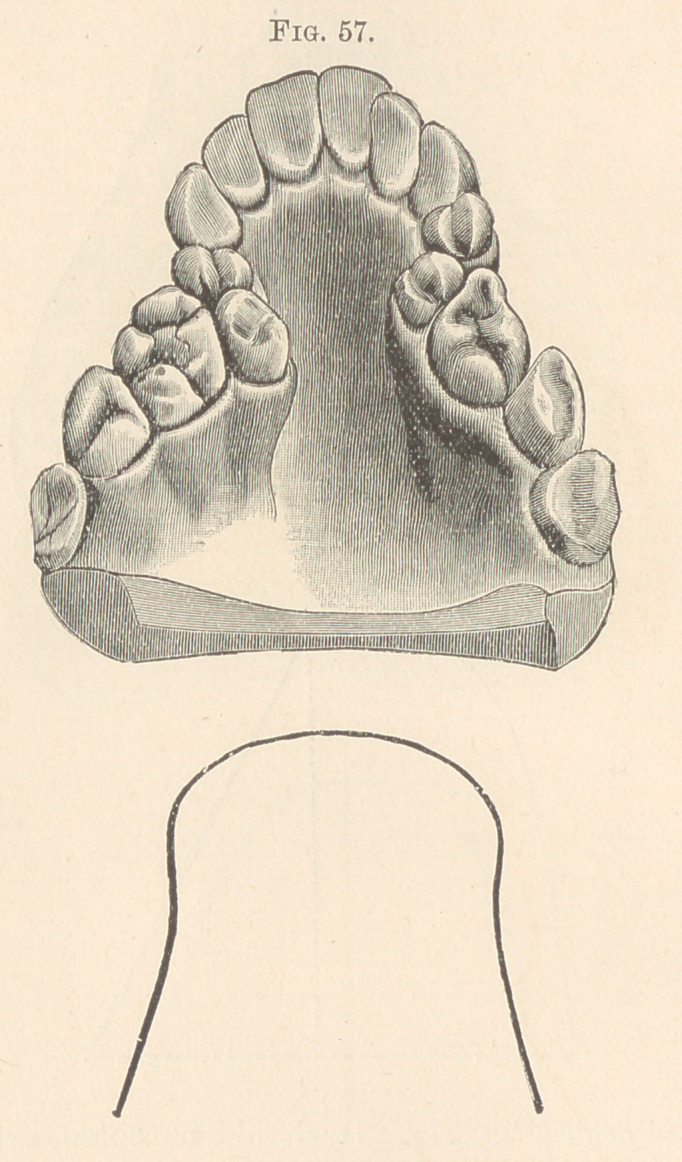


**Fig. 58. f4:**
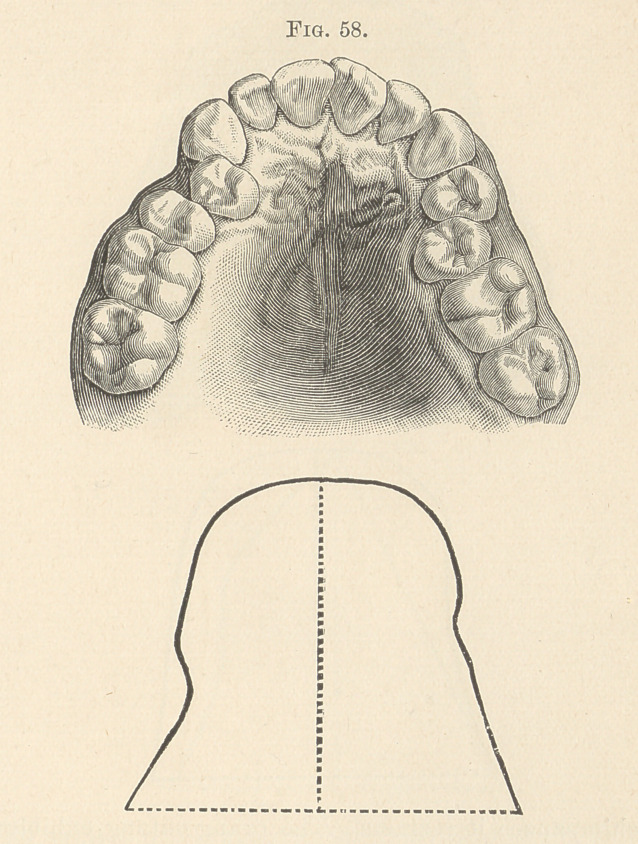


**Fig. 59. f5:**
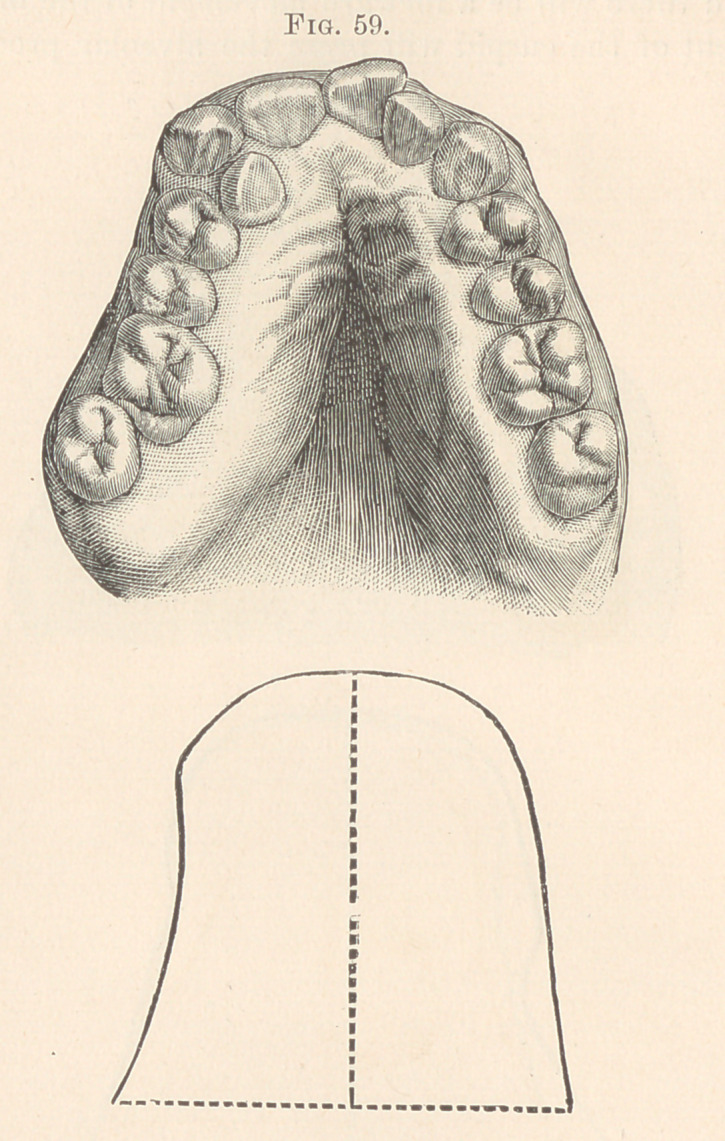


**Fig. 60. f6:**
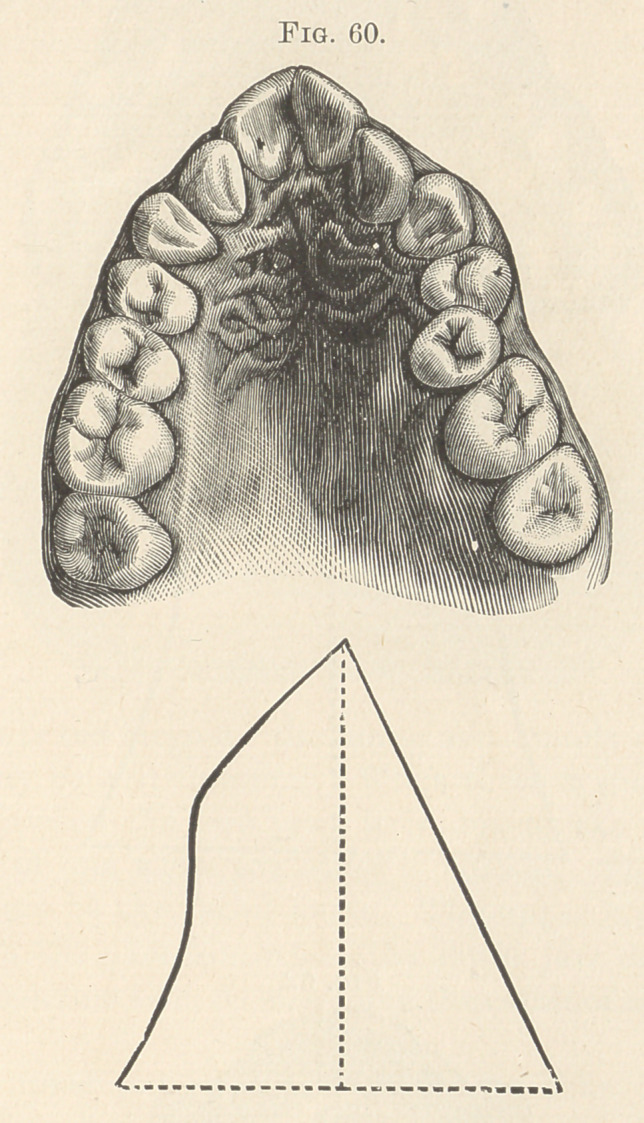


**Fig. 61. f7:**
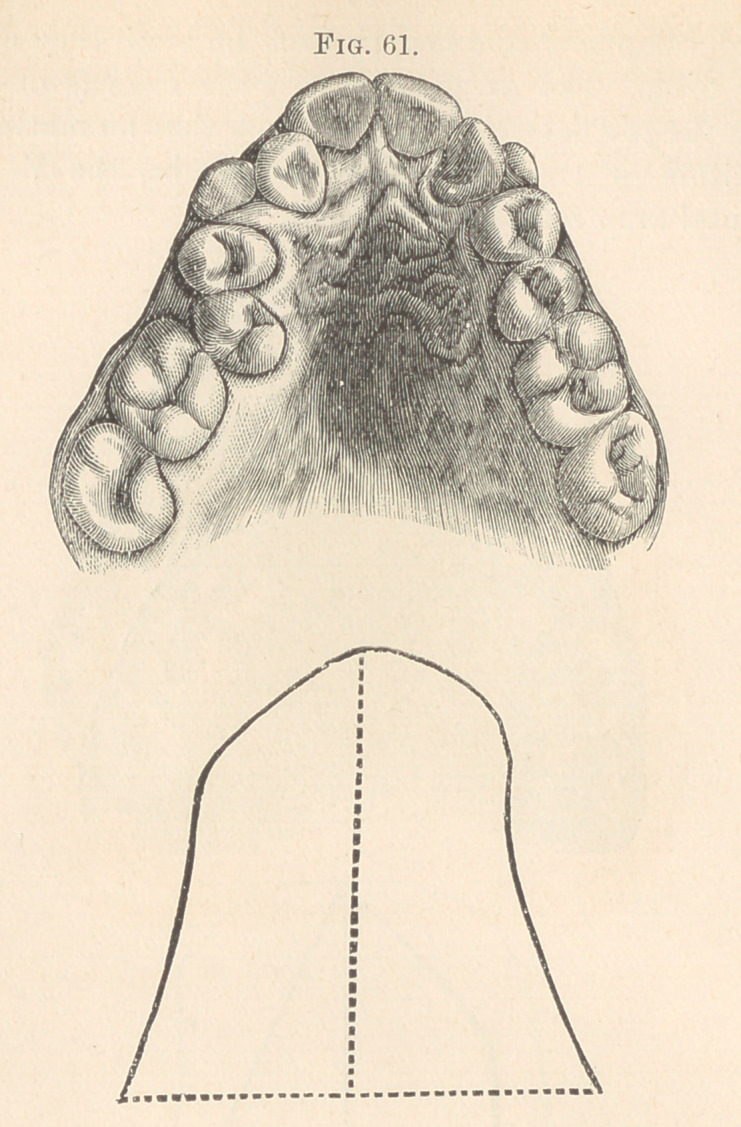


**Fig. 62. f8:**
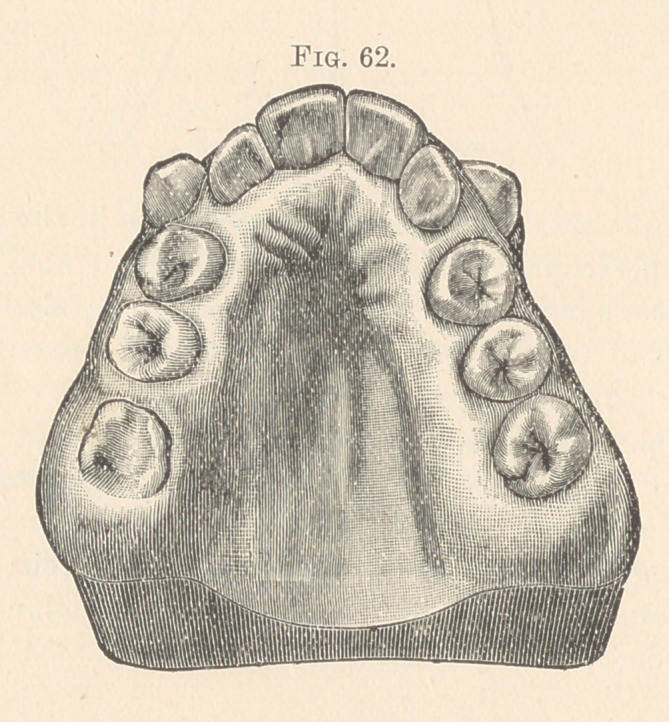


**Fig. 63. f9:**
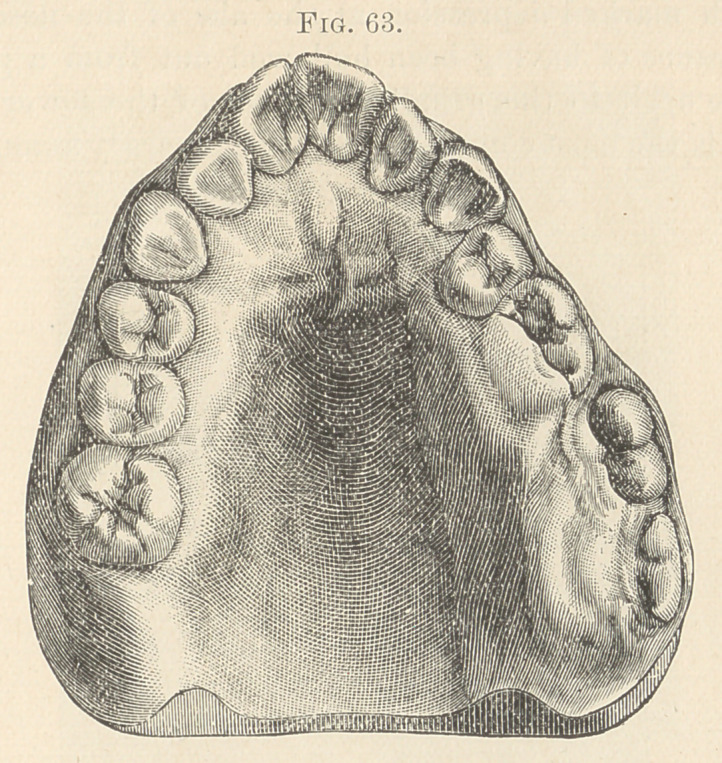


**Fig. 64. f10:**
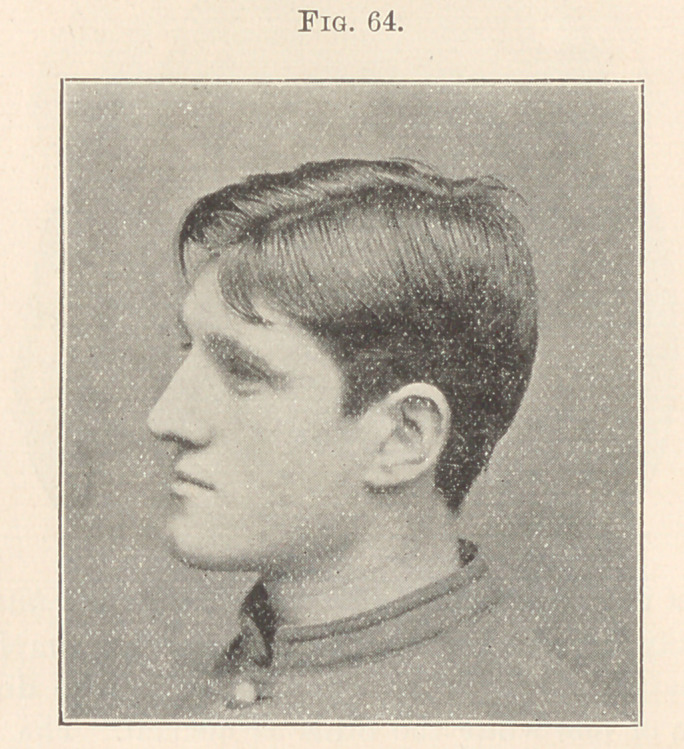


**Fig. 65. f11:**